# Identifying and quantifying the factors associated with cholera-related death during the 2018 outbreak in Nigeria

**DOI:** 10.11604/pamj.2020.37.368.20981

**Published:** 2020-12-22

**Authors:** Kelly Osezele Elimian, Anwar Musah, Chinwe Lucia Ochu, Somtochukwu Stella Onwah, Oyeronke Oyebanji, Sebastian Yennan, Ibrahima Soce Fall, Michel Yao, Martin Chukwuji, Eme Ekeng, Patrick Abok, Linda Haj Omar, Thieno Balde, Adamu Kankia, Nanpring Williams, Kitgakka Mutbam, Naidoo Dhamari, Ifeanyi Okudo, Wondimagegnehu Alemu, Clement Peter, Chikwe Ihekweazu

**Affiliations:** 1 Nigeria Centre for Disease Control, Abuja, Nigeria,; 2 University of Benin, Edo State, Nigeria,; 3 University College London, London, United Kingdom,; 4 World Health Organization/ Regional Office for Africa, Democratic Republic of Congo,; 5 World Health Organization/ Nigeria, Abuja, Nigeria

**Keywords:** Factors, cholera, case fatality rate, death, outbreak, multi-sectoral, Nigeria

## Abstract

**Introduction:**

cholera outbreaks in Nigeria are often associated with high case fatality rates; however, there is a dearth of evidence on context-specific factors associated with the trend. This study therefore aimed to identify and quantify the factors associated with cholera-related deaths in Nigeria.

**Methods:**

using a cross-sectional design, we analysed surveillance data from all the States that reported cholera cases during the 2018 outbreak, and defined cholera-related death as death of an individual classified as having cholera according to the Nigeria Centre for Disease Control case definition. Factors associated with cholera-related death were assessed using multivariable logistic regression and findings presented as adjusted odds ratios (ORs) with 95% Confidence Intervals (95% CIs).

**Results:**

between January 1 and November 19, 2018, 41,394 cholera cases were reported across 20 States, including 815 cholera-related deaths. In the adjusted multivariable model, older age, male gender, living in peri-urban areas or in flooded states, infection during the rainy season, and delay in seeking health care by >2 days were positively associated with cholera-related death; whereas living in urban areas, hospitalisation in the course of illness, and presentation to a secondary hospital were negatively associated with cholera-related death.

**Conclusion:**

cholera-related deaths during the 2018 outbreak in Nigeria appeared to be driven by multiple factors, which further reemphasises the importance of adopting a multisectoral approach to the design and implementation of context-specific interventions in Nigeria.

## Introduction

Cholera is an acute watery diarrhoea disease that is caused by the ingestion of food or water contaminated with the toxigenic strains of Vibrio cholerae serogroup O1 or O139. Cholera infection is often characterised by a rapid onset of watery diarrhoea, with or without vomiting and extensive dehydration [1]; it can also be characterised by a rapid loss of fluids if rehydration therapy is not administered promptly, potentially resulting in severe clinical sequel including lethargy, unconsciousness, confusion, drop in blood pressure and death [1]. While the case fatality rates (CFRs) from untreated cholera can be as high as 30-50%, administration of rehydration therapy has been shown to be effective, decreasing CFRs to as low as 1% [2]. Globally, deaths from cholera could be as high as 95,000 (21,000-143,000) per year [3], disproportionately affecting the most vulnerable and poorest populations with inadequate access to and preventive interventions [4]. Nigeria has been endemic for cholera since its first notification of the disease in 1970 [5]. Notable cholera outbreaks in the country with high CFRs include: 22,931 cases and 2,945 deaths (12.8% CFR) in 1971; 59,478 cases and 7,654 deaths (12.9% CFR) in 1991; 26,358 cases and 2,085 deaths (7.9% CFR) in 1999; and 41,787 cases and 1,716 deaths (4.1% CFR) in 2010 [6].

To reduce cholera-related deaths by 90% by 2030 in all the endemic countries, the Global Task Force on Cholera Control (GTFCC) and partners, in 2017, is supporting and coordinating the implementation of a multi-sectoral approach in all cholera endemic countries, including in Nigeria [7]. Indeed, Nigeria through the Nigeria Centre for Disease Control (NCDC) has taken positive steps in this direction by working collaboratively with diverse and relevant stakeholders (e.g. World Health Organization and National Primary Health Care Development Agency among others) to contextualise the global strategic framework for implementation. Furthermore, as well as successfully deploying millions of Oral Cholera Vaccines (OCVs) to cholera hotspots (predominantly in the northern region of the country), the country is making substantial progress to improving Water, Sanitation, and Hygiene (WaSH) services at various levels of governance [8].

However, the cholera outbreak in 2018 is a major public health event with deleterious impacts on sociocultural and economic activities; and indicative that attaining the global strategic goals, especially in relation to cholera-related deaths, for the country needs to be supported with context-specific research evidence. While there is evidence on the risk factors for cholera infection including inadequate WASH interventions [9], consumption of contaminated food [10], environmental and climatic conditions [11], pattern of population migration [12], increasing armed conflicts and community misconceptions [5, 13]) in Nigeria, there appears to be a paucity of evidence on what are the context-specific factors associated with adverse clinical outcomes, such as cholera-related deaths). For instance, a review of the literatures on cholera epidemiology in Nigeria found no specific evidence on the factors associated with cholera-related death [14], and a previous analysis of surveillance data from a major cholera outbreak in recent time was primarily descriptive in nature [15]. Indeed, targeting the risk factors for cholera infection for public health interventions is enormous public health benefits, but adopting a similar approach to address cholera-related death may not be effective given the variations in clinical and epidemiological outcomes. Thus, the overarching aim of this study was to fill the identified research gap by identifying and quantifying the context-specific factors associated with cholera-related death during a major outbreak in Nigeria.

## Methods

### Study design and setting

This was a cross-sectional study of national surveillance dataset during January to November 2018 in Nigeria. Nigeria comprises of 36 states and a Federal Capital Territory (FCT), and is further divided into six geopolitical zones including south-east, south-south, south-west, north-central, north-east and north-west. This study is however limited to 20 states (including the FCT) that reported cholera cases during the outbreak period. These states and their corresponding geopolitical zones include: Anambra and Ebonyi states (south-east); Adamawa, Borno, Bauchi, Gombe and Yobe states (north-east); Abuja, Kogi, Kwara, Nasarawa, Niger and Plateau states (north-central); and Jigawa, Kaduna, Kano, Katsina, Kebbi, Sokoto and Zamfara states (north-west).

### Definition of key study variables

A suspected cholera case [herein: cholera case] was defined according to the NCDC guidelines: detection of individuals aged 2 years or older with acute watery diarrhoea and severe dehydration or dying from acute watery diarrhoea from the same area within one week [16]. A confirmed cholera case was defined as a suspected case in which *Vibrio cholera* O1 or O139 was isolated in the stool. Variables (covariates) potentially associated with cholera-related deaths were identified based on evidence in the literature [17-24] as well as a consideration of biological plausibility. Table 1 summarises the definitions of key study variables.

**Table 1:** definition of key study variables

Variable	Definition and type of key variable
Cholera death	Cholera death was defined as death of a suspected cholera case. The definition was applicable to individuals who died at reporting health facilities or those who died on arrival at a reporting health facility. The variable was coded binary: alive vs dead.
Age	Age, in years, was based on the report by a patient or a primary caregiver. The decision of whether to model age as a continuous or categorical variable was however based on the outcome of Likelihood ratio test (LRT). Here, age was divided into quintiles and the model fits were compared when it was fitted as a categorical variable and not indicating it was a categorical variable. The p-value from LRT test was statistically significant (i.e. <0.0001), indicating that age was better modelled as a categorical rather than as a continuous variable. Thus, age was coded as a categorical variable: <2 years, 2-4 years, 5-10 years, 11-14 years, and 15 years or older.
Outbreak setting	Each reporting Local Government Area (LGA) was initially classified either as rural, peri-urban or urban setting. Here, we used the population division of the United Nations’ approach which classifies an urban area as a settlement with 20,000 or more inhabitants, of which 75% or more are engaged in work other than agriculture; and a rural area as a settlement with fewer than 20,000 inhabitants, of which the majority of the inhabitants are farmers. However, given the lack of a standard classification scheme for a peri-urban area, areas that were neither urban nor rural in the traditional sense were classified as peri-urban. Then, in acknowledgement of the potential limitations associated with this classification approach (e.g., increasing population growth with time), the DSNOs and/or state Epidemiologists of each reporting state were contacted by phone by the research team to validate the initial classification.
Time to health seeking	Time to health seeking, in days, was defined as the difference between reported date healthcare was sought and reported date of illness onset. It was classified as a categorical variable: same day, 1-2 days, and 2 days or more.
Hospitalization	Hospitalization was defined as a cholera case requiring admission to a formal health facility for at least one night. It was coded as a binary variable: Yes and No.
Insurgency activity (armed conflict)	Although armed conflicts could be driven by several factors, that which is perpetuated by Boko Haram insurgency in the north-eastern region of Nigeria (predominantly in in Borno, Yobe, and Adamawa states) was the primary focus for this study. These affected states directly affected by the conflict were assigned a code of “1” for insurgency activity while the remaining states were assigned a code of “0” for the absence of insurgency activity (i.e. a binary variable).
Flooding in 2018	We defined states that experienced flooding in 2018 as those declared under national disaster by the Nigeria National Emergency Management Agency (NEMA). Thus, Anambra, Kogi and Niger states were assigned a code of “1” (other states in the same category did not report cholera cases during the 2018 outbreak) while the remaining reporting states were assigned a code of “0” (i.e. a binary variable).

### Data management and statistical analyses

De-identified dataset (in MS Excel) was obtained from the Surveillance and Epidemiology Department at NCDC, managed and analysed using Stata version 15 (StataCorp LP, College Station, TX, USA). We used the ‘missing-indicator´ approach to handled missing data in order to minimise selection bias [25]. For descriptive analyses, binary and categorical variables were described using frequencies and percentages (%) in relation to cholera-related death outcome (dead vs survivor). Univariable analyses were then conducted such that the association between each covariate and cholera-related death was investigated, in turn, and presented as unadjusted odds ratios (ORs) with 95% Confidence Intervals (95% CIs). This was followed by multivariable analyses using a stepwise multiple logistic regression (backward) approach, wherein the association between cholera-related death and each statistically significant (p-value < 0.05) covariate from the univariable analyses was assessed. Here, all the variables with significant p-values in the univariable model were included in the multivariable model, and they were then removed one at a time from the model according to which is least statistically significant until those remaining in the model were statistically significant. The adjusted logistic regression model contained covariates that were significantly associated with cholera-related death, and were presented as adjusted ORs and 95% CIs.

### Ethics

The protocol for this study was reviewed and approved by the Federal Capital Territory (FCT, Nigeria) Health Research Ethics Committee (Approval Number: FHREC/2019/01/05/21-01-19).

## Results

**Figure 1:**
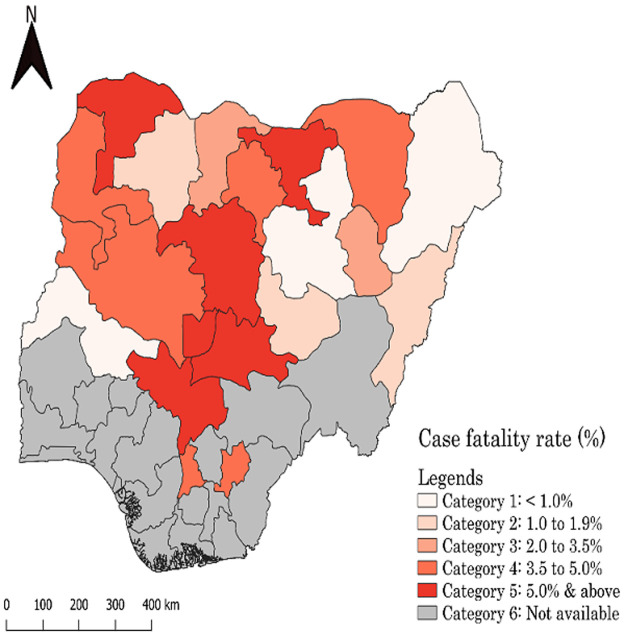
spatial distribution of case fatality rates in the 2018 cholera outbreak in Nigeria

Overall, there were 41,394 cholera cases and 815 cholera-related deaths during the study period (January 1^st^ and November 16^th^, 2018), resulting in an overall case fatality rate of 1.97% (Figure 1for the spatial distribution of cholera fatality rates). The average age of cholera cases was 21 years, with those aged 20-40 years (26.5%) and 2-5 years (19.9%) accounting for a higher proportion of deaths than the other age groups (Table 2). Males accounted for 54.7% of cholera-related deaths while females (51.3%) slightly dominated males (48.7%) with respect to the proportion of survivals. Rural areas (44.3%) accounted for the highest number of cholera-related deaths. With respect to time to health seeking following illness onset, the majority of cholera cases during the outbreak period sought health care in time, with 62.1% of those who survived survivals do so the same day of illness onset. In general, regardless of outcome status, the majority of cholera cases were hospitalised.

**Table 2:** association between the characteristics of cholera cases and clinical outcome, Nigeria, 2018 (N=41,394)

Characteristic	Clinical outcome		
	Alive (%) (n=40,579)	Dead (%) (n=815)	P-value
**Age, years†**			<0.001
2-5	9,256 (22.81)	162 (19.88)	
6-12	7,956 (19.61)	153 (18.77)	
13-19	4,771 (11.76)	66 (8.10)	
20-40	12,128 (29.89)	216 (26.50)	
41-59	2,918 (7.19)	96 (11.78)	
≥60	2,196 (5.41)	115 (14.11)	
Missing	1,354 (3.34)	7 (0.86)	
**Sex**			0.001
Female	29,831 (51.33)	369 (45.28)	
Male	19,748 (48.67)	446 (54.72)	
**Outbreak setting**			<0.001
Rural	14,533 (35.81)	361 (44.29)	
Peri-urban	5,289 (13.03)	195 (23.93)	
Urban	20,313 (50.06)	249 (30.55)	
Missing	444 (1.09)	10 (1.23)	
**Season**			<0.001
Dry	2,189 (5.39)	21 (2.58)	
Rainy	38,390 (94.61)	794 (97.42)	
**Flooding in 2018**			<0.001
No	28,372 (69.92)	469 (57.55)	
Yes	12,207 (30.08)	356 (42.45)	
**Armed conflict (Boko Haram insurgency)**			0.004
No	28,954 (71.35)	619 (75.95)	
Yes	11,625 (28.65)	196 (24.05)	
**Time to health seeking, day**			<0.001
Same day	25,217 (62.14)	445 (54.60)	
1-2	9,477 (23.35)	162 (19.88)	
>2	1,508 (3.72)	46 (5.64)	
Missing	4,377 (10.79)	162 (19.88)	
Location healthcare was sought*			<0.001
Primary healthcare centre	7,640 (18.83)	223 (27.36)	
Secondary hospital	5,595 (13.79)	66 (8.10)	
Tertiary hospital	53 (0.13)	3 (0.37)	
Private clinic	136 (0.34)	1 (0.12)	
Cholera treatment centre	672 (1.66)	5 (0.61)	
Home	6 (0.01)	3 (0.37)	
Missing	26,477 (65.25)	514 (63.07)	
**Hospitalisation**			0.002
No	5,337 (13.15)	113 (13.87)	
Yes	18,797 (46.32)	327 (40.12)	
Missing	16,445 (40.53)	375 (46.01)	
**Sample collected for rapid diagnostic test**			0.508
No	19,728 (48.62)	413 (50.67)	
Yes	1,083 (2.67)	21 (2.58)	
Missing	19,768 (48.71)	381 (46.75)	
**Rapid diagnostic test outcome**			0.575
Negative	194 (0.48)	6 (0.74)	
Positive	844 (2.08)	17 (2.09)	
Missing	39,541 (97.44)	792 (97.18)	

† Mean (Standard Deviation): 20.73(18.02); *Cholera treatment centres included camps for internally displaced persons.

In the univariable model for the odds of cholera-related death (Table 3), increasing age (with the exception of those aged 6-12 years) appeared to be associated with higher odds of cholera-related death when compared with children aged 2-5 years (reference age group)-for instance, adults classified as having cholera and aged =60 years had about three-fold higher odds of dying from cholera as compared with the reference age group (unadjusted OR 2.99,95% CI: 2.35-3.82). Regarding gender, the odds of cholera-related death was 27% higher in males than in females (unadjusted OR 1.27, 95% CI: 1.11-1.47). Also significantly (p < 0.05) associated with higher odds of cholera-related death were living in peri-urban (unadjusted OR 1.48, 95% CI: 1.24-1.77), cholera infection during the rainy season (unadjusted OR 2.16, 95% CI: 1.39-3.33), residency in states affected by flooding during the outbreak (unadjusted OR 1.71, 95% CI: 1.49-1.97), healthcare seeking >2 days after illness onset (unadjusted OR 1.72, 95% CI: 1.27-2.35), and self-report of home-based management of illness (unadjusted OR 17.13, 95% CI: 4.26-68.93). However, residency in conflict-affected states appeared protective against cholera-related death, as the odds of death from cholera was 21% lower among residents of these states than their counterparts residing in other states (unadjusted OR 0.79, 95% CI: 0.67-0.93). Additionally, living in urban areas and being hospitalised in the course of illness appeared to be negatively associated with cholera-related deaths. In the adjusted model, all but residency in conflict-affected states remained statistically significant. Specifically, after adjusting for other variables, individuals aged 41-59 years (adjusted OR 1.81,95% CI: 1.40-2.35) and ≥ 60 years (adjusted OR 2.96,95% CI: 2.31-3.79) remained positively associated with cholera-related death. The odds ratio for cholera-related death was 30% higher in males than in females (adjusted OR 1.30, 95% CI: 1.12-1.50). With respect to healthcare seeking timeline, delay by more than two days after illness onset increased the odds of cholera-related death (adjusted OR 2.08, 95% CI: 1.52-2.85). However, presentation to secondary hospitals and cholera treatment centres remained negatively associated with cholera-related death, as was hospitalisation in the course of illness.

**Table 3:** odds of cholera-related deaths in Nigeria between January and November, 2018 (n=41,394)

Variable	Cholera-related death			
	Unadjusted ORs (95% CI)	P-value	Adjusted ORs (95% CI)	P-value
**Age, years**				
6-12	1.10 (0.88-1.37)	0.408	1.08 (0.86-1.35)	0.505
13-19	0.79 (0.59-1.05)	0.110	0.82 (0.61-1.09)	0.168
20-40	1.02 (.83-1.25)	0.868	1.05 (0.85-1.30)	0.634
41-59	1.88 (1.46-2.43)	<0.001	1.81 (1.40-2.35)	<0.001
≥60	2.99 (2.35-3.82)	<0.001	2.96 (2.31-3.79)	<0.001
Missing	0.29 (0.14-0.63)	0.002	0.28 (0.13-0.62)	0.002
**Sex**				
Male	1.27(1.11-1.47)	0.001	1.30 (1.12-1.50)	0.001
**Setting**				
Peri-urban	1.48 (1.24-1.77)	<0.001	1.29 (1.06-1.57)	0.010
Urban	0.49 (0.42-0.58)	<0.001	0.58 (0.48-0.70)	<0.001
Missing	0.91 (0.48-1.71)	0.763	0.89 (0.47-1.72)	0.735
**Season**				
Rainy	2.16 (1.39-3.33)	0.001	2.50 (1.61-3.88)	<0.001
**Flooding in 2018**				
Yes	1.71 (1.49-1.97)	<0.001	1.30 (1.05-1.59)	0.014
**Armed conflict**				
Yes	0.79 (0.67-0.93)	0.004	1.04 (0.87-1.25)	0.656
**Time to health seeking, day**				
1-2	0.97 (0.81-1.16)	0.731	1.10 (0.91-1.33)	0.309
>2	1.72 (1.27-2.35)	<0.001	2.08 (1.52-2.85)	<0.001
Missing	2.10 (1.74-2.52)	<0.001	2.16 (1.77-2.63)	<0.001
**Location healthcare was sought**				
Secondary hospital	0.40 (0.31-0.53)	<0.001	0.55 (0.41-0.76)	<0.001
Tertiary hospital	1.93 (0.60-6.25)	0.268	2.04 (0.62-6.72)	0.241
Private clinic	0.25 (0.04-1.81)	0.171	0.33 (0.05-2.43)	0.279
Cholera treatment centre (including IDP camps)	0.25 (0.10-0.62)	0.003	0.47 (0.19-0.87)	0.006
Home	17.13 (4.26-68.93)	< 0.001	21.17 (5.09-88.12)	<0.001
Missing	0.67 (0.57-0.78)	< 0.001	0.99 (0.79-1.24)	0.939
**Hospitalisation**				
Yes	0.82 (0.66-1.02)	0.075	0.57 (0.45-0.72)	<0.001
Missing	1.08 (0.87-1.33)	0.494	0.76 (0.60-0.96)	0.020
**Sample collected for rapid diagnostic test**				
Yes	0.93 (0.60-1.44)	0.734		
Missing	0.92 (0.80-1.06)	0.249		
**Rapid diagnostic test outcome**				
Positive	0.65 (0.25-1.67)	0.373		
Missing	0.65 (0.29-1.46)	0.296		

## Discussion

The study has identified and quantified context-specific factors associated with cholera-related death during a major cholera outbreak in Nigeria. Specifically, these factors ranged from demographic (older age, and male gender), environment or residential setting, to healthcare capacity, practices and seeking behaviours. The association between a setting (rural, peri-urban and urban) and cholera-related death could be attributable, in part, to variations in infrastructural development (including health facilities) and social amenities, as well as access to and delivery of health care services in Nigeria. Historically, rural areas in Nigeria tend to be less developed in terms of health care system and social amenities as compared to urban areas. Moreover, despite less work load and better willingness of community members to adhere to public health recommendations, health professionals in rural areas tend to be less motivated to work given inadequate social and health amenities [26]. This could negatively contribute to healthcare delivery by health professionals and seeking by community members in such settings. However, the higher odds of cholera-related death among peri-urban residents as compared to rural residents could be attributable to predominance of cholera transmission drivers in such settings. For instance, poor drainage networks and high population density characteristic of peri-urban areas in Nigeria are known risk factors for adverse clinical outcomes among cholera patients [27]. The improved availability of trained and motivated health professionals as well as essential kits for case management and WASH indicators in urban areas [28] may possibly explain the decreased odds of death from cholera infection among urban residents in the current study. Further supporting our explanations is the higher concentration of critical infrastructures for health care services, including specialised cholera treatment centres, in urban than in rural areas in Nigeria [29]. Thus, as well as improving the required medical materials to health workers in rural areas, our findings underline the need to increase investments on integrated training of primary health care workers in such settings in Nigeria. While acknowledging the potential for our definition of settings to be biased given the classifications based on DSNO´s/state epidemiologists´ knowledge of their locality, these findings could be of benefit to policy makers and researchers in prioritising areas for interventions and research activities. This is because current cholera hotspots in Nigerian States, as identified by several criteria including CFRs from 2012 to 2017 [30], may not be granular enough to capture cholera epidemiology in contrasting settings.

Compared with prompt health seeking (i.e. same day of illness onset), prolonging health care by more than two days doubled the risk of cholera-related death in the current study. Studies in Malawi [17], Cameroon [18] and Guinea-Bissau [19] have reported a similar trend. This pattern of association is not surprising given the short incubation period (1.4 days) [31] of *V. cholera* with the capacity to result in severe dehydration and death in the absence of appropriate care [22, 32]. Given the public health implications of delay in health care following an illness onset, further investigation, preferably using a qualitative study approach, aimed at understanding the contextual barriers to health seeking is warranted in the Nigerian setting. Findings from such an in-depth study could be potentially useful designing appropriate and sustainable public health interventions against cholera-related death in the country. This thinking is supported by a study in Zimbabwe where implementation of an intervention based on this framework resulted in substantial decrease in cholera deaths [23]. We found also that cholera infection during the rainy season appeared to have increased the odds of cholera-related death in comparison to dry season; and by 30% in flooded states when compared with non-flooded states. These could be explained by increased precipitation during the rainy season, often characterised by flooding, especially in the context of poor drainage networks [27]; and by obstruction of essential health care services (e.g. disruption of essential health services, relief aids, and disruption of transportation system). Thus, there is a need to expand public health collaborations, especially between the National Emergency Management Agency and Centre for Disease Control in preparing for and responding to cholera outbreaks in Nigeria. This multi-sectoral approach is at the core of the GTFCC´s global strategic goals for cholera endemic countries [7].

Older age groups (49-59 years and ≥ 60 years) were more at a higher risk of death from cholera infection children aged 2-5 years. Children are generally more prone to death from cholera infection due to difficulty in the clinical management (e.g. fluid management) of paediatric cholera cases as well as their higher susceptibility to malnutrition [17] which exacerbates the risk of cholera-related death in endemic areas [2]. Nonetheless, the current finding could be attributable, in part, to a decrease in immunity with increasing age, especially given the limited and incomplete oral cholera vaccination coverage in the country as at December 2017. This finding however underline the need to expand acute clinical care beyond children to include adult during an outbreak, and prioritisation of persons for OCVs. Although similar findings have been reported in Haiti [22], Zimbabwe [23], and Ethiopia [24], the higher odds of cholera-related death in males as compared to their female counterparts needs to be investigated further considering the unique socio-cultural parameters in Nigeria.

This study is contributing to fill an important gap in the literature, and providing evidential-basis to inform the design and implementation of public health interventions aimed at mitigating severe clinical outcomes among cholera patients in the Nigerian context. In addition, findings from this study are relatively more generalisable to the cholera-affected states during the outbreak as all the analysed data arose from a national public health database. The study however has a number of limitations worth mentioning. First, it is possible for cases classified as cholera-related deaths to have been omitted given the reliance on surveillance data based on a non-random screening approach. For instance, exploring alternative data sources (e.g. burial records) can yield more data on cholera-related death than routine surveillance database [33], albeit our data source did capture events at the community level in the course of active contact tracing. Second, our definition of cholera implies that some cholera-related deaths could be misclassified given that cholera-like symptoms can be caused by pathogens other than *V. cholera* [34]; conversely, deaths attributable to other pathogens might have been misclassified as cholera-related deaths. Moreover, only a few diagnostic tests (e.g. RDT) were available in the data source for this study, thus our findings need to be interpreted with caution. Third, the linelist utilised for this study was limited in terms of its scope given the lack of some essential variables-e.g. pregnancy status, childhood malnutrition, degree of dehydration etc.-known to be independently associated with cholera-related death [22, 35]. This highlights one of the challenges associated with using secondary datasets for research purposes, particularly in developing settings.

## Conclusion

Findings in the current study suggest that cholera-related deaths in Nigeria during the 2018 outbreak were mainly driven by multiple factors ranging from health systems, demographics, environmental to socio-behavioural factors. These findings further underline the importance of adopting a multi-sectoral approach to the design and implementation of public health interventions against adverse clinical outcomes among cholera patients.

### What is known about this topic

- Cholera is endemic in Nigeria with high case fatality rates;
- Evidence on the factors associated with cholera infection in Nigeria is available.

### What this study adds

- The study has identified context-specific factors associated with cholera-related death during a major cholera outbreak in Nigeria;
- Multiple factors are associated with cholera-related death in Nigeria, re-emphasising the importance of a multisectoral approach to designing and implementing interventions.
